# Dihydroartemisinin Attenuated Intervertebral Disc Degeneration via Inhibiting PI3K/AKT and NF-*κ*B Signaling Pathways

**DOI:** 10.1155/2022/8672969

**Published:** 2022-09-09

**Authors:** Zhiheng Liao, Deying Su, Hengyu Liu, Caixia Xu, Jinna Wu, Yuyu Chen, Weimin Guo, Shun Zhang, Zhuling Li, Xiaona Ke, Tingting Wang, Taifeng Zhou, Peiqiang Su

**Affiliations:** ^1^Department of Spine Surgery, Guangdong Provincial Key Laboratory of Orthopedics and Traumatology, The First Affiliated Hospital of Sun Yat-sen University, Guangzhou 510080, China; ^2^Guangdong Provincial Key Laboratory of Proteomics, State Key Laboratory of Organ Failure Research, Department of Pathophysiology, School of Basic Medical Sciences, Southern Medical University, Guangzhou 510515, China; ^3^Research Centre for Translational Medicine, The First Affiliated Hospital of Sun Yat-Sen University, Guangzhou 510080, China

## Abstract

Intervertebral disc degeneration (IDD) is the leading cause of low back pain (LBP). However, effective therapeutic drugs for IDD remain to be further explored. Inflammatory cytokines play a pivotal role in the onset and progression of IDD. Dihydroartemisinin (DHA) has been well reported to have powerful anti-inflammatory effects, but whether DHA could ameliorate the development of IDD remained unclear. In this study, the effects of DHA on extracellular matrix (ECM) metabolism and cellular senescence were firstly investigated in nucleus pulposus cells (NPCs) under tumor necrosis factor alpha (TNF*α*)-induced inflammation. Meanwhile, AKT agonist sc-79 was used to determine whether DHA exerted its actions through regulating PI3K/AKT and NF-*κ*B signaling pathways. Next, the therapeutic effects of DHA were tested in a puncture-induced rat IDD model. Finally, we detected the activation of PI3K/AKT and NF-*κ*B signaling pathways in clinical degenerative nucleus pulposus specimens. We demonstrated that DHA ameliorated the imbalance between anabolism and catabolism of extracellular matrix and alleviated NPCs senescence induced by TNF*α in vitro*. Further, we illustrated that DHA mitigated the IDD progression in a puncture-induced rat model. Mechanistically, DHA inhibited the activation of PI3K/AKT and NF-*κ*B signaling pathways induced by TNF*α*, which was undermined by AKT agonist sc-79. Molecular docking predicted that DHA bound to the PI3K directly. Intriguingly, we also verified the activation of PI3K/AKT and NF-*κ*B signaling pathways in clinical degenerative nucleus pulposus specimens, suggesting that DHA may qualify itself as a promising drug for mitigating IDD.

## 1. Introduction

Low back pain (LBP) is an extremely common symptom worldwide and an estimated 80% of adults experience LBP at least once in their lifetime [1, 2]. LBP causes incredible economic burden on the society [3]. Intervertebral disc degeneration (IDD) is the leading cause of LBP and contributes to 40% of LBP [4–6]. Despite the high prevalence and huge economic burden of IDD, it is urgent to further elucidate its molecular mechanism [7, 8]. Intervertebral disc (IVD) consists of the outer annulus fibrosus (AF), the central gelatinous nucleus pulposus (NP) and cartilaginous endplates, and it is embedded between the vertebrae, providing flexibility to the spine and enabling spinal motion. NP is mainly composed of nucleus pulposus cells (NPCs) and surrounding extracellular matrix (ECM), secreted by NPCs [9]. And the degeneration of NP is generally considered as the initiating factor of IDD [10].

Most IDD begins in adulthood and progresses with age, and the pathologies of IDD are complex. Besides age-related changes during IDD [11], multiple factors have been well documented, including degradation of extracellular matrix (ECM) [10], overproduction of inflammatory cytokines [12], NPC senescence [13], and excessive mechanical stretch [14]. NPCs are the major cell type in NP and play a pivotal role in maintaining the homeostasis of ECM. The integrity of ECM depends on two main constituents, including the type I and type II collagen (COL2A1) network, and water-binding proteoglycans such as aggrecan (ACAN) [9]. During the progression of IDD, synthesis of COL2A1 and ACAN is downregulated and ECM degradative molecules such as matrix metalloproteinase (MMP), and a disintegrin and metalloproteinases with thrombospondin motifs (ADAMTS), are exceedingly upregulated [15]. Inadequate anabolism and excessive catabolism lead to the destruction of ECM [10].

Apart from ECM degradation, inflammatory cytokines have emerged as a prominent role in the development of IDD [16, 17]. It is well documented that tumor necrosis factor *α* (TNF*α*) plays a pivotal role in IDD [12, 18], involving in multiple pathological changes of disc degeneration [12]. TNF*α* strongly induces the expression of MMPs and ADAMTS, leading to the irreversible ECM destruction, NPCs senescence, and deterioration of the IVDs microenvironment [19, 20]. Additionally, TNF*α* exerts formidable proinflammatory activity, inducing IL-1*β*, IL-6, IL-8, and inhibiting IL-10, which triggers inflammatory cascades and further enhances the ECM degradation [21, 22]. Taken together, TNF*α* contributes to the occurrence of IDD and LBP. Therefore, anti-TNF therapy has a promising future in the treatment of IDD and LBP [23].

Cellular senescence is a permanent cell cycle arrest triggered by various stimuli, characterized by upregulation of p16 and p21, enhanced *β*-galactosidase (*β*-gal) activity, and senescence-associated secreted phenotype (SASP) [24, 25]. And SASP is featured by increased secretion of proinflammatory cytokines, chemokines, and proteases. Cellular senescence is causally linked to the development of IDD [26]. NPCs senescence has been confirmed in human degenerative NP tissues by *β*-gal staining and contributes to IDD progression [27]. Senescent NPCs have lower ECM production capabilities and accelerate senescence in the neighboring cells through SASP [26]. Through *Cdkn2a* (encoding p16^Inka4^) knockout mouse, several teams have demonstrated that p16Ink4a deletion inhibits SASP and cell senescence and ameliorates the progression of IDD [28–30]. Thus, antisenescence therapy might shed light on the treatment of IDD.

Dihydroartemisinin (DHA), the active metabolite of artemisinin, has a more powerful antimalarial effect and is widely used as the first-line antimalarial drug. In addition, A large body of evidence suggests that DHA exerts immunomodulation, antitumor, and anti-inflammation pharmacological effects [39], especially anti-inflammation. For instance, DHA ameliorates LPS-induced acute lung, kidney injury, and neuroinflammation by depressing inflammation [31, 32, 37]. However, whether DHA exerts a protective effect against IDD remains to be elucidated.

In this study, we demonstrated that DHA ameliorated the imbalance of ECM metabolism, alleviated NPCs senescence induced by TNF*α in vitro*, and mitigated the IDD progression in a puncture-induced rat model. And DHA exerted its protective effects by regulating PI3K/AKT and NF-*κ*B signaling pathways. Intriguingly, the activation of the above signaling pathways was confirmed in our clinical degenerative nucleus pulposus specimens, suggesting that DHA may qualify itself as a promising drug for mitigating IDD by targeting the above signaling pathways.

## 2. Materials and Methods

### 2.1. Reagents and Antibodies

DHA was purchased from MCE (NJ, USA). Recombinant human TNF*α* was purchased from Novoprotein (Shanghai, China). Cell counting Kit-8 (CCK-8) was purchased from Glpbio (CA, USA). The cell culture reagents were purchased from Gibco (NY, USA).

The primary antibodies against COL2A1, ACAN, MMP13, MMP3, ADAMTS4, ADAMTS5, p21, and p16 were purchased from Abcam (Cambridge, England), and the primary antibodies against MMP9, iNOS, COX2, NF-*κ*B p65, p-NF-*κ*B p65, PI3K, p-PI3K, AKT, p-AKT, p-IKB*α*, IKB*α*, IKK, p-IKK, and GAPDH were purchased from Cell Signaling Technology (Boston, USA). Secondary antibodies were obtained from Invitrogen (CA, USA) and Proteintech (Chicago, USA). ELISA kits for IL-1*β*, IL-6, IL-8, and IL-10 were purchased from Boster (CA, USA).

### 2.2. Human Samples

Human NP tissues were collected, preserved, and tested according to the protocols and guidelines of the Medical Ethics Committee of the First Affiliated Hospital of Sun Yat-sen University (Guangzhou, China) (No. [2022]033). Eighteen clinical specimens were included in this study from July 15th to October 27th. Pathological tissues were removed from patients with disc herniation during spinal surgery, and control tissues were removed from patients with lumbar fractures or idiopathic scoliosis. All patients were informed of all the surgical risks, and informed consents were signed before the surgery. Patient information is shown in Table 1. The severity of disc degeneration was graded according to Pfirrmann's grading system [33]. Specimens of grade I-II were included in control group (*n* = 4), and specimens of grade III were included in mild group (*n* = 7), while specimens of grade IV-V were included in severe group (*n* = 7).

### 2.3. Culture of NPCs

Human NPCs were purchased from ScienCell (Carlsbad, USA) and cultured in nucleus pulposus cell medium supplemented with 10% FBS (Gibco), 1% NPC growth supplement (Gibco), and 1% penicillin/streptomycin (Gibco). The cells were cultured in a humidified incubator at 37°C with 5% CO2. NPCs were treated with different concentrations of DHA (500 and 1000 nM, MCE) and TNF*α* (10 ng/mL, Novoprotein).

### 2.4. Cell Viability Assay

**Figure 1:**
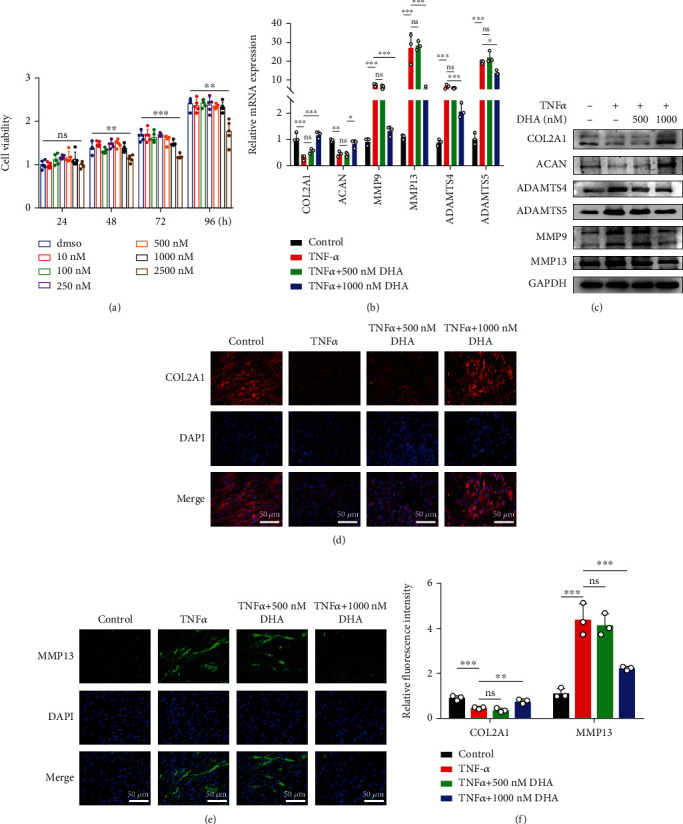
DHA ameliorated the metabolic imbalance of NPCs under TNF*α*-induced inflammation. (a) Cytotoxic effect of different concentrations of DHA in NPCs was detected by CCK-8 assay at indicated time points. (b) Relative mRNA expression of *COL2A1*, *ACAN*, *ADAMTS4*, *ADAMTS5*, *MMP9*, and *MMP13* was detected by qPCR in TNF*α*-treated NPCs with or without DHA. (c) Protein expression of COL2A1, ACAN, ADAMTS4, ADAMTS5, MMP9, and MMP13 was detected by western blotting in TNF*α*-treated NPCs with or without DHA. (d–e) The expression of COL2A1 (d) and MMP13 (e) was detected by immunofluorescence combined with DAPI indicating the nuclei. Scale bar =50 *μ*m. (f) The fluorescence intensity of COL2A1 and MMP13 was evaluated by Image J software. The values are presented as means ± SD (*n* = 3). ^∗^*P* < 0.05, ^∗∗^*P* < 0.01, ^∗∗∗^*P* < 0.001.

NPCs were seeded in 96-well plates at a density of 5,000 cells per well for 24 h. Then, NPCs are treated with different concentrations of DHA as shown in Figure 1(a) and cultured for 0, 24, 48, 72, and 96 h. At the indicated time, 100 *μ*L fresh medium with 10 *μ*L CCK-8 (Glpbio) was added to each well. After incubated for 2 h, the absorbance of 96-well plates was measured by a microplate reader (Tecan Sunrise, Salzburg, Austria) at 450 nm. The cell viability was calculated according to the manufacturer's instruction.

### 2.5. Real-Time Quantitative PCR

Total RNA was extracted using the TRIzol reagent (Invitrogen, CA, USA) according to the manufacturer's protocol. An amount of 500 nanograms of total RNA was reverse transcribed to synthesize cDNA using NovoScript® 1st Strand cDNA Synthesis Kit (Novoprotein, Shanghai, China). qPCR was performed using NovoStart® SYBR qPCR SuperMix Plus (Novoprotein, Shanghai, China) on a Roche LightCycler 96 Detection System (Roche, Vienna, Austria). The primer sequences are listed in Table 2. The relative mRNA expression was calculated by the 2^-△△Ct^ method and normalized to *GAPDH* expression [34].

### 2.6. Western Blot Analysis

Total protein was extracted using RIPA Lysis Buffer (Beyotime, Shanghai, China) containing 1% protease inhibitor and phosphatase inhibitor (Bimake, TX, USA). Equal amounts of protein samples were subjected to 10% or 15% SDS-PAGE (Solarbio, Beijing, China), separated by electrophoresis, and transferred to nitrocellulose filter membranes. The membranes were blocked with 5% skim milk powder for 1 h at room temperature, and then, the target membranes were incubated at 4°C overnight with primary antibodies against COL2A1, ACAN, ADAMTS4, ADAMTS5, MMP3, MMP13, p16 or p21 (1 : 1000, Abcam), ADAMTS5 (1: 250, Abcam), MMP9, iNOS, COX2, NF-*κ*B p65, p-NF-*κ*B p65, AKT, p-AKT, p-PI3K, PI3K, p-IKB*α*, IKB*α*, p-IKK or IKK (1 : 1000, Cell Signaling Technology), or GAPDH (1 : 5000, Proteintech). After incubated with HRP-conjugated secondary antibody for 1 h at room temperature, the signal was developed using an ECL chemiluminescence detection kit (Beyotime, Shanghai, China), and images were captured on ImageQuant Las4000mini (GE Healthcare, Tokyo, Japan).

### 2.7. Immunofluorescence

NPCs were seeded on coverslips and treated with indicated agents for 48 h. Then, the slips were fixed with 4% paraformaldehyde (Biosharp, Hefei, China) for 15 min, permeabilized with 0.5% Triton-100 (Solarbio, Beijing, China) for 5 min, and blocked with 10% goat serum (Boster, CA, USA) for 30 min, followed by incubation at 4°C overnight with primary antibodies against COL2A1, MMP13, MMP3 (1 : 100, Abcam), and NF-*κ*B p65 (1 : 100, Cell Signaling Technology). Next day, the slips were incubated with Alexa Fluor®-conjugated secondary antibodies for 1 h at room temperature, followed by incubation with DAPI (Solarbio, Beijing, China) for 5 min. The images were captured with a fluorescence microscope (Olympus BX63, Tokyo, Japan), and the fluorescence intensity was evaluated through software Image J.

### 2.8. Senescence-Associated *β*-Galactosidase (SA-*β*-Gal) Staining

*β*-gal staining (Beyotime, Beijing, China) was carried out according to the manufacturer's protocol. Briefly, the cells were fixed in fixative at room temperature for 15 min and incubated with working solution at 37°C without CO_2_ overnight. The images were captured with an inverted microscope, and the ratio of *β*-gal^+^ cells was calculated.

### 2.9. Puncture-Induced Rat IDD Model

A total of 24 adult female Sprague-Dawley rats (12 weeks old) were purchased from Charles River Laboratory (Beijing, China). All experimental procedures were approved by Institutional Animal Care and Use Committee of Sun Yat-sen University (No. SYSU-IACUC-2021-000973). All rats were randomly divided into three groups: the control group, the IDD group, and the IDD + DHA group, and raised under 12 h light/dark conditions with free access to water and food. The IDD model was established as described before [35].

Briefly, the rats were anaesthetized, and tail skin was sterilized, and then, needles (21G) were adopted to puncture the NP to a depth of approximately 5 mm and rotated 360° and kept in the disc for 1 min. After the surgery, the rats of control and IDD groups were injected intraperitoneally with DMSO, while the rats of IDD + DHA group were treated with 40 mg/kg DHA (dissolved in DMSO) every other day.

### 2.10. Magnetic Resonance Imaging (MRI)

Four weeks after surgery, the disc degeneration of all rats was examined by MRI. Briefly, the rats were anaesthetized, and the tails were straightened. T2-weighted spin-echo sequences in the sagittal and horizontal planes were adopted to detect the discs of coccyx. The degeneration of discs was evaluated by Pfirrmann's grading system as previously described [33].

### 2.11. Histological Analysis

The freshly dissected rat tails were fixed overnight in 4% paraformaldehyde, decalcified, and embedded in paraffin. Then, the specimens were cut into 5-*μ*m thickness sections. The sections were dewaxed, rehydrated, and stained with hematoxylin and eosin (H&E) and safranin O-fast green (SO). For H&E staining, the sections were stained with hematoxylin for 1 minute and differentiated in 0.5% acid alcohol for two seconds, followed by eosin counterstain for 2 minutes. After dehydration in alcohol and clearance in xylene, the sections were mounted with resins and glass coverslip. As for SO staining, the sections were stained with 0.02% fast green for 5 minutes and differentiated in 1% acetic acid for 30 seconds, followed by 1% safranin O counterstain for 2 minutes. After dehydration in alcohol and clearance in xylene, the sections were mounted with resins and glass coverslip. The images were captured with a microscope (Leica DMI4000B, Wetzlar, Germany).

### 2.12. Immunohistochemistry

Tissue sections were dewaxed, rehydrated, and performed heat-mediated antigen retrieval. The sections were incubated at 4°C overnight with primary antibodies against COL2A1, ACAN, MMP13, TNF*α* (1 : 100, Abcam), p-PI3K (1 : 100, Cell Signaling Technology), NF-*κ*B p65 (1 : 250, Proteintech), p16 (1 : 250, Bioss), and IL6 (1 : 200, Abcam). Then, the sections were incubated with HRP-conjugated secondary antibody, and the signal was developed with 3,3′-diaminobenzidine. The images were captured with a microscope (Leica DMI4000B, Wetzlar, Germany). Image-Pro Plus version 6.0 (Media Cybernetics, Inc., Rockville, MD, USA) was used to evaluate the dyed area and the integrated optical density (IOD), and mean density equaled the IOD value divided by area. The calculation was executed by two observers blinded to the experimental design.

### 2.13. Molecular Docking

The molecule 3D structure file (7k6m.pdb) of PI3K was obtained from the PDB database (https://www.rcsb.org/). Dihydroartemisinin molecule 3D structure file was obtained from the NCBI PubChem Compound database (https://pubchem.ncbi.nlm.nih.gov/). 7k6m.pdb was opened using AUTODOCK TOOLS (version 1.5.6, Scripps Research Institute, CA, USA); ligand residues, water molecules, and ions were removed; and polar hydrogen atoms and Kollman charges were added and saved as 7k6m.pdbqt. The docking pocket was defined near the ligand binding pocket. The dihydroartemisinin structure file was opened using PyMOL (version 2.4, Schrodinger LLC., NY, USA) and saved as a pdb format file. Gasteiger charge was added to dihydroartemisinin structure using AUTODOCK TOOLS, and a pdbqt format file of dihydroartemisinin structure was stored. After preparing the receptor and ligand files and related configuration files, the ligand molecules were docked into the set receptor pocket using AUTODOCK VINA software (Scripps Research Institute, California, US), and the top 10 molecular models were output.

### 2.14. Statistical Analysis

The results from at least three independent experiments were presented as the mean ± standard deviation. All data were analyzed using SPSS 20.0 statistical software package (SPSS, Inc., Chicago, IL, USA), and the statistical significance was determined by unpaired Student's *t*-test between two groups or by one-way analysis of variance (ANOVA) followed by Tukey's multiple comparison test among more than two groups. The *P* value less than 0.05 was considered statistically significant.

## 3. Results

### 3.1. Cytotoxicity of DHA in NPCs

Various DHA concentrations, ranging from 10 nM to 100 *μ*M, were used according to different cell types [31, 32, 36, 37], but no toxic effects of DHA on NPCs have yet been reported in previous literature. The maximum blood concentration of DHA for malaria in clinical practice is 0.71 mg/L, equivalent to 2500 nM. Therefore, we set up a concentration gradient of DHA (0, 10, 100, 250, 500, 1000, and 2500 nM) to treat NPCs and then determined the toxic effects via CCK-8 assay at 24, 48, 72, and 96 h after treatment. We observed that cell viability was significantly inhibited under 2500 nM DHA administration (Figure 1(a)), so concentrations of 500 nM and 1000 nM were chosen for subsequent experiments.

### 3.2. DHA Ameliorated the Metabolic Imbalance of NPCs under TNF*α*-Induced Inflammation

We next explored the effects of DHA on the matrix metabolism of NPCs under TNF*α*-induced inflammation by qPCR, western blotting, and immunofluorescence. The qPCR and western blotting results indicated that TNF*α* suppressed the expression of COL2A1 and ACAN, which were the main constituents of ECM, and 1000 nM DHA, but not 500 nM DHA, ameliorated the suppression. Meanwhile, 1000 nM DHA, instead of 500 nM DHA, inhibited ECM catabolism markers induced by TNF*α*, such as ADAMTS4, ADAMTS5, MMP9, and MMP13 (Figures 1(b) and 1(c)). The metabolism changes were further confirmed by immunofluorescence staining of COL2A1 and MMP13. Consistently, 1000 nM DHA reversed the changes in MMP13 and COL2A1 induced by TNF*α* (Figures 1(d)–1(f)).

### 3.3. DHA Ameliorated NPCs Senescence Induced by TNF*α*

**Figure 2:**
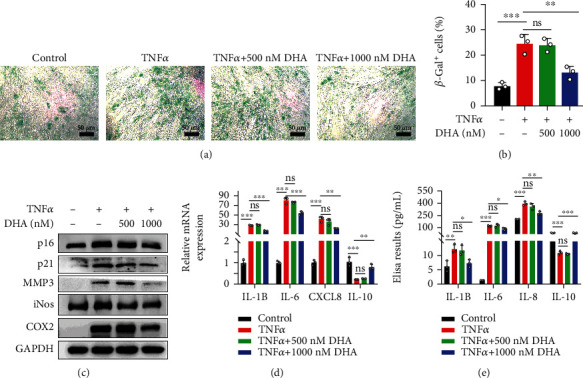
DHA ameliorated NPCs senescence induced by TNF*α*. (a) Cellular senescence of TNF*α*-treated NPCs with or without DHA was detected by *β*-Gal staining. Scale bar =50 *μ*m. (b) Quantitative analysis of the rate of *β*-Gal^+^ senescent NPCs in (a). (c) Protein expression of senescence markers (p16 and p21) and SASP markers (MMP3, iNOS, and COX2) was detected by western blotting in TNF*α*-treated NPCs with or without DHA. (d) Relative mRNA expression of SASP markers (*IL-1B*, *IL-6*, and *CXCL8*) and *IL-10* was detected by qPCR in TNF*α*-treated NPCs with or without DHA. (e) Concentrations of SASP markers (IL-1B, IL-6, and IL-8) and IL-10 in the cell culture medium of TNF*α*-treated NPCs with or without DHA, were determined by ELISA. The values are presented as means ± SD (*n* = 3). ^∗^*P* < 0.05, ^∗∗^*P* < 0.01, ^∗∗∗^*P* < 0.001.

During the development of IDD, the accumulation of senescent NPCs accelerates IDD progression. Recently, it is reported that TNF*α* could enhance SA-*β*-gal activity as well as the expression of senescence markers (p16 and so on), thus promoting NPC senescence [18]. We further explored whether DHA could alleviate NPC senescence induced by TNF*α*. Our results indicated that TNF*α* intensively enhanced SA-*β*-gal activity and promoted the expression of senescence markers (p16 and p21), which was reversed by 1000 nM DHA, but not 500 nM DHA (Figures 2(a)–2(c)). Further, we detected whether DHA could improve TNF*α*-induced SASP in NPCs by qPCR, western blotting, and ELISA. As shown in Figure 2(c), TNF*α* induces the expression of MMP3, iNOS, and COX2, which were inhibited by 1000 nM DHA, but not 500 nM DHA. Furthermore, our results showed that TNF*α* upregulated the expression and secretion of IL-1B, IL-6, and IL-8, and downregulated IL-10, and 1000 nM DHA reversed the changes induced by TNF*α*, but not 500 nM DHA (Figures 2(d) and 2(e)). Together, these data suggested that DHA ameliorated NPCs senescence induced by TNF*α*.

### 3.4. DHA Suppressed the NF-*κ*B and PI3K/AKT Signal Pathways in TNF*α*-Treated NPCs

**Figure 3:**
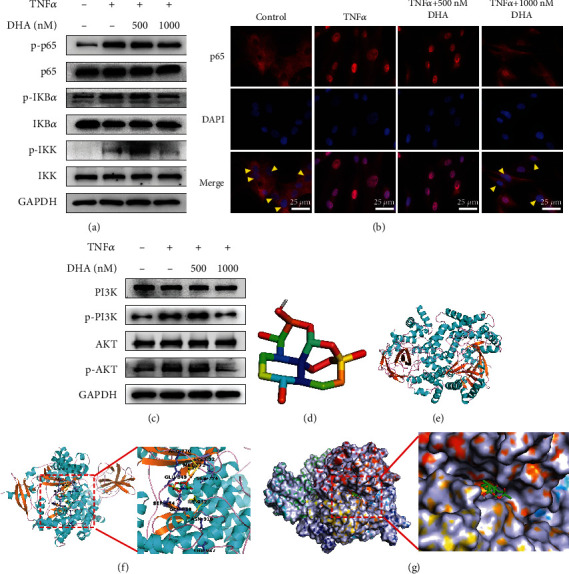
DHA suppressed the NF-*κ*B and PI3K/AKT signal pathways in TNF*α*-treated NPCs. (a) Western blotting of NF-*κ*B pathway proteins in TNF*α*-treated NPCs with or without DHA for 72 hours. (b) Nuclear translocation of NF-*κ*B p65 (red) was determined by immunofluorescence staining in TNF*α*-treated NPCs with or without DHA for 30 min. DAPI (blue) indicated nuclei. Yellow arrowheads indicated cytoplasmic distribution of NF-*κ*B p65. Scale bar =25 *μ*m. (c) Western blotting of PI3K pathway proteins in TNF*α*-treated NPCs with or without DHA for 72 hours. (d) Model of DHA. (e) Ribbon model of PI3K. (f–g) Space filling model between DHA and PI3K and reaction between DHA and PI3K.

To explore the underlying mechanism of DHA protecting NPCs under inflammation, we first detected the classical downstream pathway of TNF*α* by western blotting and immunofluorescence staining. As shown in Figure 3, TNF*α* significantly activates NF-*κ*B signal pathway by upregulating the ratio of p-NF-*κ*B p65/NF-*κ*B p65, p-IKB*α*/IKB*α*, and p-IKK/IKK compared to the control group, which could be reversed by 1000 nM DHA, but not 500 nM DHA (Figure 3(a)). Besides, immunofluorescence results showed that TNF*α* promoted nuclear translocation of NF-*κ*B p65 which was inhibited by 1000 nM DHA, but not 500 nM DHA (Figure 3(b)). These results showed that DHA inhibited the activation of NF-*κ*B signal pathway induced by TNF*α*.

To probe the upstream signal pathway of NF-*κ*B, we detected whether PI3K/AKT signal pathway was involved in the effects of TNF*α*-treated NPCs by western blotting. Upon the treatment of TNF*α*, the levels of phosphorylation of PI3K and AKT were upregulated, which was reversed by 1000 nM DHA, but not 500 nM DHA (Figure 3(c)). Several reports have demonstrated that DHA has a beneficial effect on inflammatory diseases via the PI3K/AKT signal pathway [36, 38]. To determine whether DHA binds directly to PI3K, molecular docking between DHA and PI3K was carried out. Top ten models are obtained, and interaction energy is listed in Table 3. We found that DHA had a good connection with PI3K in the “pocket,” as shown in Figure 3(g). Besides, amino acids within 2.2 Å of DHA included hydrophobic amino acids MET-777 and ILE-932 and hydrophilic amino acids LYS-802, GLU-849, SER-774, ARG-770, SER-854, GLN-859, ASN-918, and THR-957 (Figure 3(f)). These results suggested that DHA suppressed PI3K/AKT and NF-*κ*B signal pathways in TNF*α*-treated NPCs and had a direct binding with PI3K.

### 3.5. AKT Agonist Sc-79 Undermined the Protective Effects of DHA against IDD In Vitro

**Figure 4:**
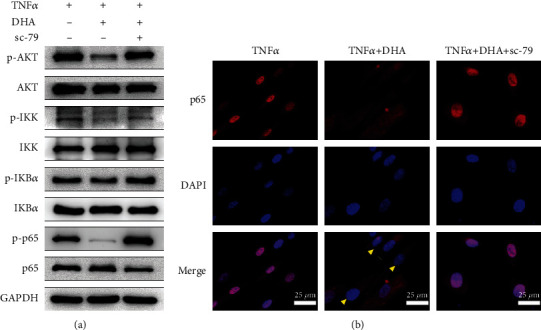
AKT agonist sc-79 undermined the suppression of DHA on PI3K/AKT and NF-*κ*B signal pathways. (a) Western blotting of PI3K/AKT and NF-*κ*B pathway proteins under indicated treatment for 30 min. (b) Nuclear translocation of NF-*κ*B p65 (red) was determined by immunofluorescence staining under indicated treatment for 30 min. DAPI (blue) indicated nuclei. Yellow arrowheads indicated cytoplasmic distribution of NF-*κ*B p65. Scale bar =25 *μ*m.

**Figure 5:**
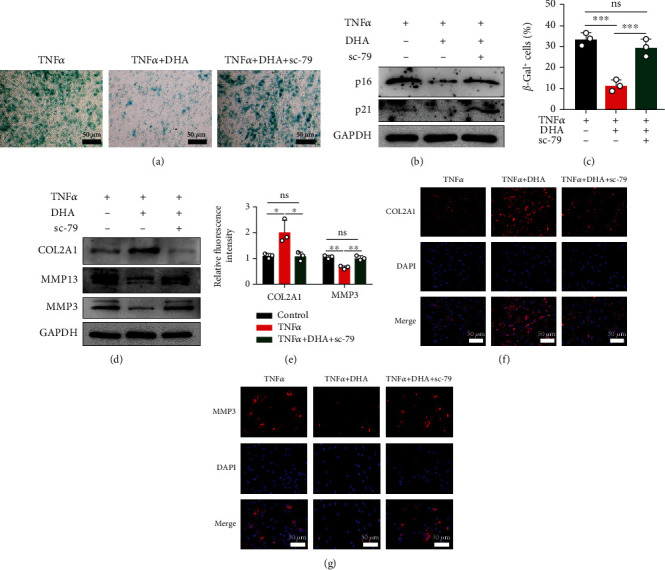
AKT agonist sc-79 undermined the beneficial effects of DHA on NPCs. (a) Cellular senescence was detected by *β*-Gal staining under indicated treatment for 48 hours. Scale bar =50 *μ*m. (b) Western blotting of senescence markers (p16 and p21) under indicated treatment for 48 hours. (c) Quantitative analysis of the rate of *β*-Gal+ senescent NPCs in (a). (d) Protein expression of COL2A1, MMP3, and MMP13 was detected by western blotting under indicated treatment for 48 hours. (e) The fluorescence intensity of COL2A1 and MMP3 was evaluated by Image J software. (f–g) The expression of COL2A1 (f) and MMP3 (g) was detected by immunofluorescence combined with DAPI staining for the nuclei. Scale bar =50 *μ*m. The values are presented as means ± SD (*n* = 3). ^∗^*P* < 0.05, ^∗∗^*P* < 0.01, ^∗∗∗^*P* < 0.001.

Recently, it is reported that NF-*κ*B signal pathway is downstream of the PI3K/AKT signal pathway during the development of IDD [39]. To confirm that PI3K/AKT regulated the NF-*κ*B signal pathway, we treated NPCs with sc-79, an agonist of AKT. We found that sc-79 undermined the suppression of DHA on PI3K/AKT and NF-*κ*B signal pathways (Figure 4(a)) and weakened the inhibition of DHA on NF-*κ*B p65 nuclear translocation (Figure 4(b)). Furthermore, we assessed whether sc-79 could undermine the beneficial effects of DHA on NPCs and our results indicated that sc-79 enhanced SA-*β*-gal activity as well as the expression of senescence markers (p16 and p21), thus weakening the alleviation of DHA on cellular senescence (Figures 5(a)–5(c)). Meanwhile, sc-79 undermined the beneficial effects of DHA on ECM metabolism through promoting the expression of MMP3 as well as MMP13 and inhibiting synthesis of COL2A1 (Figure 5(d)). Immunofluorescence staining of COL2A1 and MMP3 revealed the consistent results (Figures 5(e) and 5(f)).

### 3.6. DHA Mitigated IDD Progression *In Vivo*

**Figure 6:**
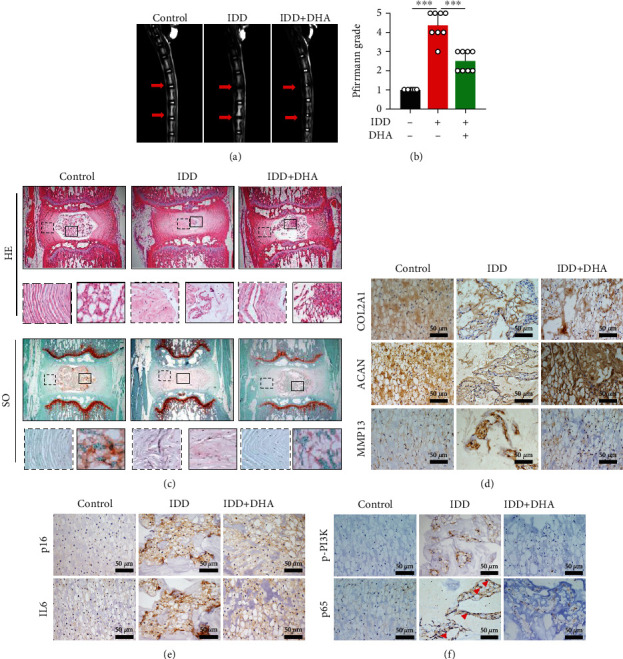
DHA mitigated IDD progression in the puncture-induced rat model. (a) Representative T2-weighted MRI images of the caudal intervertebral discs with indicated treatment. Red arrows indicated the punctured discs. (b) Pfirrmann score analysis of T2-weighted MRI images of different groups (*n* = 8). (c) Representative images of H&E and SO staining of different groups. The areas marked by solid and dashed boxes were magnified below the corresponding images. (d–f) Immunohistochemical staining for COL2A1, ACAN, and MMP13 (d); p16 and IL6 (e); and p-PI3K and NF-*κ*B p65 (f) in rat intervertebral discs of different groups. Red arrowheads indicated nuclear translocation of NF-*κ*B p65. Scale bar =50 *μ*m. The values are presented as means ± SD (*n* = 8). ^∗^*P* < 0.05, ^∗∗^*P* < 0.01, ^∗∗∗^*P* < 0.001.

To investigate whether DHA could ameliorate the progression of IDD *in vivo*, we established puncture-induced rat model. The degeneration of discs was detected by magnetic resonance imaging (MRI) and scored by Pfirrmann's grade system. In the control group, the tail discs showed high intensity of T2-weighed MRI signal, while the IDD group showed significantly low intensity, with a higher Pfirrmann score. Administration of DHA could significantly rescue the intensity of water signal and lower Pfirrmann scores (Figures 6(a) and 6(b)). Further, we harvested the tail discs and detected changes of the histology. In the control group, round-shaped NP with evenly distributed nuclear cells, well-organized collagen lamellas and a clear boundary between AF and NP were observed. However, in the IDD group, a great loss of nuclear cells, disorganized lamellas, and interrupted border was observed. The administration of DHA significantly alleviated the disc degeneration (Figure 6(c)).

Moreover, the immunohistochemical staining results showed that compared to the control group, upregulation of MMP13, along with downregulation of COL2A1 and ACAN, was observed in the IDD group which was reversed by administration of DHA (Figure 6(d)). Additionally, the markers of cellular senescence, p16 and IL-6, were increased in the IDD group, whereas DHA could inhibit the levels of p16 and IL-6 (Figure 6(e)). Furthermore, DHA could also decrease the levels of p-PI3K and inhibit the nuclear translocation of NF-*κ*B p65 (Figure 6(f)). Together, DHA mitigated IDD progression *in vivo*, and it may be associated with inhibition of PI3K/AKT and NF-*κ*B signal pathways.

### 3.7. Activation of PI3K/AKT and NF-*κ*B Signal Pathways in Degenerative NP Tissues

**Figure 7:**
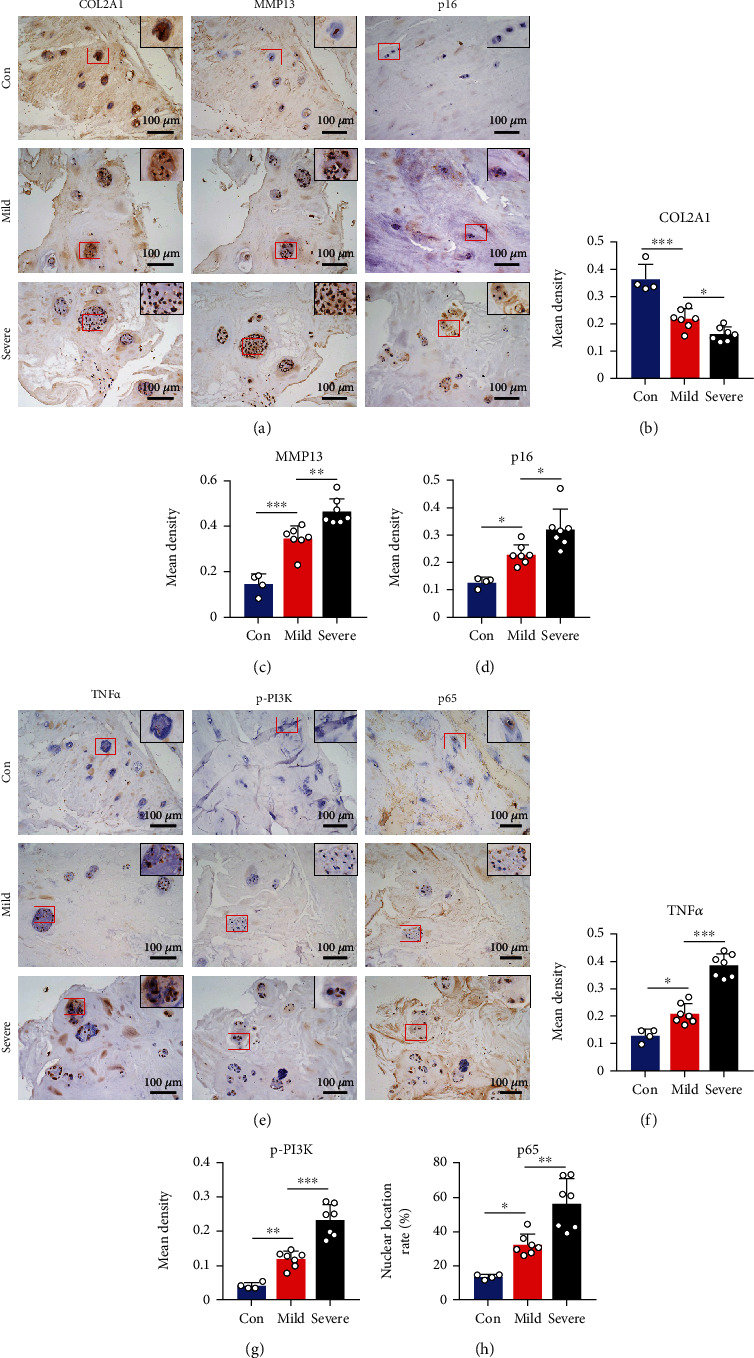
Activation of PI3K/AKT and NF-*κ*B signal pathways in degenerative NP tissues. (a) Immunohistochemical staining for COL2A1, MMP13, and p16 in clinical NP tissues of different degeneration degrees. The areas marked by red boxes were magnified at upper right corner of the corresponding images. Scale bar =100 *μ*m. (b–d) Mean density of IOD values of COL2A1 (b), MMP13 (c), and p16 (d) in clinical NP tissues of different degeneration degrees. (e) Immunohistochemical staining for TNF*α*, p-PI3K, and NF-*κ*B p65 in clinical NP tissues of different degeneration degrees. The areas marked by red boxes were magnified at upper right corner of the corresponding images. Scale bar =100 *μ*m. (f–g) Mean density of IOD values of TNF*α* (f) and p-PI3K (g) in clinical NP tissues of different degeneration degrees. (h) Nuclear translocation rate of NF-*κ*B p65 per field in clinical NP tissues of different degeneration degrees. The values are presented as means ± SD. Con group *n* = 4, mild group *n* = 7, and severe group *n* = 7. ^∗^*P* < 0.05, ^∗∗^*P* < 0.01, ^∗∗∗^*P* < 0.001.

To provide more evidence for the therapeutic potential of DHA, we assessed the alteration of PI3K/AKT and NF-*κ*B signal pathways in clinical degenerative NP tissues. We first confirmed the imbalance of ECM metabolism in degenerative NP tissues along with the increase of NPCs senescence (Figures 7(a)–7(d)). Moreover, TNF*α* was substantially expressed in mild and severe IDD specimens compared to the control specimens. Simultaneously, along with the elevated expression of TNF*α*, rose the expression of p-PI3K and nuclear translocation of NF-*κ*B p65 (Figures 7(e)–7(h)). These results suggested a significant role of TNF*α* and activation of PI3K/AKT and NF-*κ*B signaling pathways in the progression of IDD. Inhibition of PI3K/AKT and NF-*κ*B signaling pathways might qualify DHA as a promising therapeutic drug in IDD treatment.

## 4. Discussion

**Figure 8:**
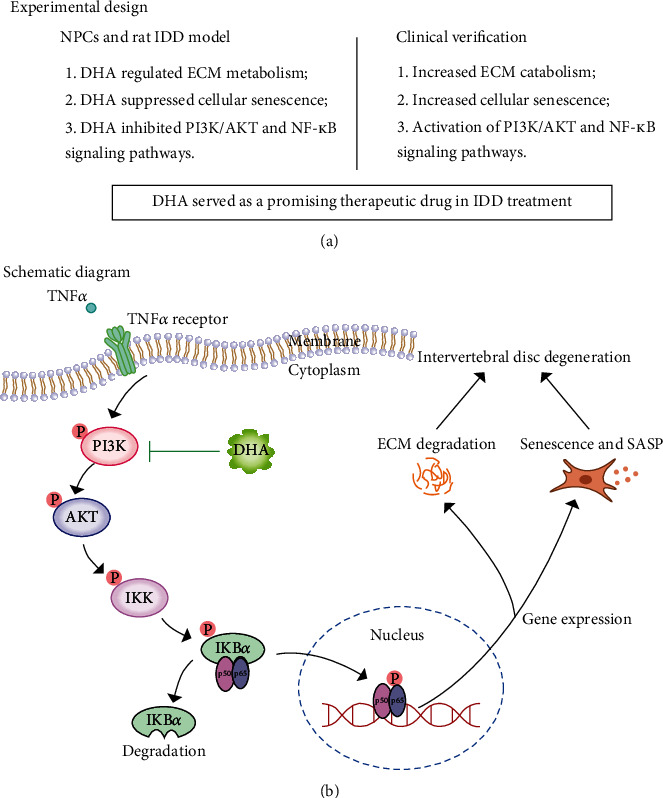
Experimental design and schematic diagram of protective effects of DHA on IDD. (a) Experimental design composed of two parts: cellular and animal experiments to verify the protective effects of DHA and clinical specimens to verify IDD pathological changes and the activation of corresponding signaling pathways. (b) DHA improved ECM metabolism and mitigated NPCs senescence via inhibiting PI3K/AKT and NF-*κ*B signaling pathways in the progression of IDD.

A large body of evidence suggests that IDD is the leading cause of LBP, which is now the main cause of disability worldwide [1]. Thus, exploring new drugs for IDD treatment is urgent. Recently, many scholars have focused on traditional Chinese drugs, such as quercetin [35], naringin [40], and karacoline [41]. DHA, derivative of artemisinin, is extracted from Chinese herb Artemisia annual. Besides its antimalarial activity, DHA has been reported to possess potent anti-inflammatory effects [39]. However, the potential role of DHA on IDD remains to be discovered. In this study, we demonstrated that DHA ameliorated IDD via inhibiting PI3K/AKT and NF-*κ*B signaling pathways and DHA might serve as a promising therapeutic drug for the treatment of IDD (Figure 8).

Notwithstanding the fact that the pathology of IDD is not fully elucidated, substantial evidence has shown that inflammatory cytokines play a critical role in the development of IDD [42], particularly TNF*α*. TNF*α* is highly expressed in IDD nucleus pulposus tissue and positively correlates with the degree of degeneration and the degree of discogenic pain [43, 44]. It has been well documented that TNF*α* not only induces the expression of MMPs and ADAMTSs to accelerate the degradation of ECM, but also promotes NPC senescence and upregulates the proinflammatory cytokines, thus worsening the microenvironment of IVDs and aggravating IDD [18, 42]. Therefore, TNF*α* is adopted to mimic inflammation in the present study.

Apart from antimalarial efficacy, DHA has been reported to have potent anti-inflammation activity and protective effects on diseases characterized by inflammation, such as neuroinflammation [36], acute kidney injury [31], colitis [38], and osteoarthritis [37], which implies that DHA has the potential therapeutic effects on IDD. Therefore, we tested the effects of DHA on IDD through *in vitro* and *in vivo* experiments. Firstly, we determined the safe concentration of DHA on NPCs and adopted 500 nM as well as 1000 nM concentrations in the following *in vitro* experiments. Next, we confirmed that DHA ameliorated the imbalance of ECM metabolism, by promoting synthesis of COL2A1 and inhibiting the expression of catabolic markers, MMPs and ADAMTSs, under TNF*α*-induced inflammation. Similarly, Jiang et al. revealed that DHA inhibited TNF*α*-induced expressions of MMP3 and MMP9 and ADAMTS5 in rat chondrocytes [37]. These results implied that DHA was instrumental in ameliorating the imbalance between anabolism and catabolism of ECM, which is beneficial to the mechanical function of the disc.

In parallel to ECM degradation, cellular senescence is causally linked to the development of IDD [14, 26]. Several studies have demonstrated that TNF*α* enhances *β*-gal activity and expression of senescence markers and promotes NPCs senescence [45–47]. In this study, we illustrated that DHA could alleviate TNF*α*-induced NPCs senescence and improve the SASP, and further in DHA-treated IDD rat model, NPCs senescence was improved by the administration of DHA. Similarly, Li et al. demonstrated that DHA inhibited myeloid-derived suppressor cells senescence to ameliorate the development of systemic lupus erythematosus [48]. Moreover, it is well documented that DHA significantly inhibits expression of proinflammatory cytokines and alleviates inflammatory diseases, including inflammatory bowel disease, rheumatoid arthritis, lupus nephritis, and allergic asthma [49]. Correspondingly, we demonstrated that DHA exerted anti-inflammation effects and improved SASP through inhibiting the expression of MMPs and proinflammatory cytokines.

Activation of the NF-*κ*B signaling pathway plays a pivotal role in TNF*α*-induced inflammation. Our results indicated that DHA inhibited the NF-*κ*B signaling pathway via lowering the ratio of p-NF-*κ*B p65/NF-*κ*B p65 and reduced the nuclear translocation of NF-*κ*B p65. Similarly, other studies have shown that DHA inhibits NF-*κ*B signaling pathway in the treatment of colitis [38], acute lung injury [32], and acute kidney injury [31]. In agreement with previous study, TNF*α* activated PI3K/AKT signaling pathway in NPCs, while DHA inhibited the phosphorylation levels of PI3K and AKT under TNF*α*-induced inflammation. Meanwhile, molecular docking indicated that DHA bound to PI3K directly, suggesting that DHA might interact with PI3K and influence the phosphorylation of PI3K with the presence of TNF*α*. It is widely accepted that NF-*κ*B signaling pathway is downstream of the PI3K/AKT signaling pathway in IDD [39]. And cytokines-induced activation of the PI3K/AKT signaling pathway is related to the NF-*κ*B signaling pathways in several cell types [50–52]. Correspondingly, our results indicated that the treatment of AKT agonist sc-79 activated NF-*κ*B signaling pathway in NPCs and undermined the protective effects of DHA. Taken together, our results revealed that DHA suppressed the effects of TNF*α* in NPCs via inhibiting PI3K/AKT and NF-*κ*B signaling pathways.

Further, we confirmed that DHA attenuated the progression of IDD in a puncture-induced rat model via inhibiting PI3K/AKT and NF-*κ*B signaling pathways. More meaningfully, we verified high expression of TNF*α* and the activation of PI3K/AKT and NF-*κ*B signaling pathways in NP tissues from clinical IDD patients, which suggested that DHA might have the potential to combat IDD in clinical practice. Our study revealed the theoretical and experimental basis for the clinical application of DHA by illustrating the mechanism underlying its protective effects against IDD. This discovery might shed light on clinical treatment of IDD. However, pharmacologic action of DHA on inflammatory disease mainly has been limited to basic or animal studies. The administration of DHA in IDD treatment during clinical practice still has a long way to go.

There are several limitations in our study. First, the interaction between DHA and PI3K is predicted by computer algorithm, which needs to be further verified by fluorescence- or biotin-labelled DHA. Second, AKT agonist sc-79 should be administrated in rat IDD model to confirm that DHA attenuated IDD via inhibiting PI3K/AKT and NF-*κ*B signaling pathways *in vivo*, which is our next step. Last but not least, clinical trials should be designed to confirm the protective effects of DHA on IDD in clinical practice.

## 5. Conclusions

In summary, we demonstrated that DHA attenuated the development of IDD via inhibiting PI3K/AKT and NF-*κ*B signal pathways. Furthermore, we confirmed the activation of PI3K/AKT and NF-*κ*B signaling pathways in degenerative nucleus pulposus tissue. In consequence, DHA might serve as a promising drug for IDD by targeting the above signaling pathways.

## Figures and Tables

**Table 1:** Details of human nucleus pulposus specimens.

Case	Gender	Age	Affected IVD	Pfirrmann grade
1	Male	41	L4/5	I
2	Female	65	L5/S1	I
3	Male	20	L3/4	I
4	Male	58	L5/S1	II
5	Female	55	L4/5	III
6	Male	53	L4/5, L5/S1	III
7	Male	63	C3/4/5	III
8	Female	48	C4/5/6	III
9	Male	50	L4/5	III
10	Female	71	L5/S1	III
11	Female	68	L4/5	IV
12	Male	53	L5/S1	IV
13	Female	63	L5/S1	IV
14	Male	59	L4/5	IV
15	Male	69	L4/5	IV
16	Female	69	L1-5	IV
17	Female	64	L4/5	V
18	Male	64	C3/4/5	V

Affected IVD, L: lumbar, S: sacral, C: cervical.

**Table 2:** Primers for qPCR.

Gene	Former primer (5′-3′)	Reverse primer (5′-3′)
*GAPDH*	AATGGGCAGCCGTTAGGAAA	GCCCAATACGACCAAATCAGAG
*COL2A1*	TGGACGATCAGGCGAAACC	GCTGCGGATGCTCTCAATCT
*ACAN*	ACTCTGGGTTTTCGTGACTCT	ACACTCAGCGAGTTGTCATGG
*ADAMTS4*	GAGGAGGAGATCGTGTTTCCA	CCAGCTCTAGTAGCAGCGTC
*ADAMTS5*	GAACATCGACCAACTCTACTCCG	CAATGCCCACCGAACCATCT
*MMP9*	GGGACGCAGACATCGTCATC	TCGTCATCGTCGAAATGGGC
*MMP13*	CCAGACTTCACGATGGCATTG	GGCATCTCCTCCATAATTTGGC
*IL1B*	ATGATGGCTTATTACAGTGGCAA	GTCGGAGATTCGTAGCTGGA
*IL6*	ACTCACCTCTTCAGAACGAATTG	CCATCTTTGGAAGGTTCAGGTTG
*CXCL8*	TTTTGCCAAGGAGTGCTAAAGA	AACCCTCTGCACCCAGTTTTC
*IL10*	TCAAGGCGCATGTGAACTCC	GATGTCAAACTCACTCATGGCT

**Table 3:** Affinity between DHA and PI3K in top ten models of molecular docking assay.

Mode	Affinity(kcal/Mol)
1	-7.4
2	-7.1
3	-6.8
4	-6.8
5	-6.7
6	-6.5
7	-6.5
8	-6.5
9	-6.4
10	-6.4

## Data Availability

The data used to support the findings of this study are available from the corresponding author upon request.
